# Socioeconomic Determinants of Bullying in the Workplace: A National Representative Sample in Japan

**DOI:** 10.1371/journal.pone.0119435

**Published:** 2015-03-09

**Authors:** Kanami Tsuno, Norito Kawakami, Akizumi Tsutsumi, Akihito Shimazu, Akiomi Inoue, Yuko Odagiri, Toru Yoshikawa, Takashi Haratani, Teruichi Shimomitsu, Ichiro Kawachi

**Affiliations:** 1 Department of Hygiene, Wakayama Medical University, Wakayama, Japan; 2 Department of Mental Health, Graduate School of Medicine, The University of Tokyo, Tokyo, Japan; 3 Department of Public Health, Kitasato University School of Medicine, Kanagawa, Japan; 4 Department of Mental Health, University of Occupational and Environmental Health, Fukuoka, Japan; 5 Department of Preventive Medicine and Public Health, Tokyo Medical University, Tokyo, Japan; 6 Department of Research, The Institute for Science of Labour, Kawasaki, Japan; 7 Health Administration and Psychosocial Factor Research Group, National Institute of Occupational Safety and Health, Kanagawa, Japan; 8 Department of Social and Behavioral Sciences, Harvard School of Public Health, Boston, United States of America; Old Dominion University, UNITED STATES

## Abstract

Bullying in the workplace is an increasingly recognized threat to employee health. We sought to test three hypotheses related to the determinants of workplace bullying: power distance at work; safety climate; and frustration related to perceived social inequality. A questionnaire survey was administered to a nationally representative community-based sample of 5,000 residents in Japan aged 20–60 years. The questionnaire included questions about employment, occupation, company size, education, household income, and subjective social status (SSS). We inquired about both the witnessing and personal experience of workplace bullying during the past 30 days. Among 2,384 respondents, data were analyzed from 1,546 workers. Multiple logistic regression analyses were used to examine the social determinants of workplace bullying. Six percent and 15 percent of the total sample reported experiencing or witnessing workplace bullying, respectively. After adjusting for gender and age, temporary employees (Odds Ratio [OR]: 2.45 [95% Confidence Interval (CI) = 1.03–5.85]), junior high school graduates (OR: 2.62 [95%CI: 1.01–6.79]), workers with lowest household income (OR: 4.13 [95%CI:1.58–10.8]), and workers in the lowest SSS stratum (OR: 4.21 [95%CI:1.66–10.7]) were at increased risk of experiencing workplace bullying. When all variables were entered simultaneously in the model, a significant inverse association was observed between higher SSS and experiencing bullying (p = 0.002). Similarly in terms of witnessing bullying; SSS was significantly inversely associated (p = 0.017) while temporary employees reported a significantly higher risk of witnessing bullying compared to permanent workers (OR: 2.25 [95%CI:1.04 to 4.87]). The significant association between SSS and experiencing/witnessing workplace bullying supports the frustration hypothesis. The power distance hypothesis was also partly supported by the finding that temporary employees experienced a higher prevalence of workplace bullying.

## Introduction

Workplace bullying is increasingly recognized as a serious public health issue in the workplace, due to both its high prevalence as well as its adverse impact on employee health [1]. The prevalence of workplace bullying has been reported to be as high as 15.7% on average in European countries, except Scandinavia [2]. A similarly high prevalence (9.0–15.5%) has been found in Asian countries including Japan [3–5]. Workplace bullying is associated with serious health problems for the victims, including psychological distress [6], depression [7,8], cardiovascular disease [7], and sickness absences [9].

Socially disadvantaged groups in the working population are at heightened risk of being victimized by workplace bullying. Three separate theoretical reasons have been put forward to explain this phenomenon. First, workplace bullying is an expression of the power distance between the perpetrator and the victim [10,11]. People with less authority in an organization are vulnerable to becoming to the target of bullying by a person with higher authority who chooses to abuse their power. Workplace bullying is thus expected to be more prevalent among occupations with lower authority (such as manual workers) compared to occupations with higher authority (such as managers and professionals). Previous studies reported a higher prevalence of workplace bullying among unskilled workers who tend to be located in the lowest rungs of organizational hierarchies; conversely, the prevalence has been reported to be lowest among managers or supervisors [12–14]. However the results are not entirely consistent; the study by Hoel et al. [15] reported that the prevalence of bullying was similar across all occupational status groups.

A different kind of power distance is expressed by the distinction between permanent workers and non-regular workers. The globalization of trade and its attendant demand for “labor flexibility” has resulted in an increase in the demand for non-regular (sometimes referred to as “precarious”) employment throughout industrialized economies. Non-regular employees are in a lower position in an organization, and often work for less pay, lack benefits (such as pensions or protection by labor laws), and they experience greater job insecurity than permanent employees [16,17]. Temporary workers ("Haken shain") represent one category of non-regular work. They consist of workers who are dispatched from agencies to work in organizations on a temporary basis. In addition to their lower position in an organization, dispatched workers are often seen as “someone from the outside”. Particularly in the context of Japanese culture—which is strongly group-oriented—the temporary worker is at risk of being doubly distanced from his peers, both in terms of the inferiority of his social status within the organization, but also in terms of the distinction between outsiders versus insiders [18].

A second determinant of workplace bullying is the safety climate within the organization. According to Leymann, organizational factors are the major causes of bullying [11,19,20], and a number of studies confirmed that workplace bullying tends to be more prevalent in workplaces with poor work environment [20–22]. Psychosocial safety climate, which is defined as the “organizational policies, practices, and procedures for the protection of worker psychosocial health and safety” [23] is a predictor of workplace bullying [24]. In Japan, larger companies are more likely to have in place formal policies, practices, and procedures for the protection of worker psychosocial health, and hence company size has been used as a proxy for safety climate [25,26]. Due to their larger budgets, bigger companies are more likely to take thorough countermeasures against workplace bullying or harassment, and they more likely to enforce compliance [27]. Based on the foregoing, we hypothesize that the prevalence of workplace bullying will tend to be lower among larger companies.

The third explanation for workplace bullying is based on the “frustration hypothesis”, viz. those who occupy a lower social position in social hierarchies are apt to experience more frustration as a result of being “pushed around” by those at the top, and/or possibly as the result of psychological feelings of insecurity stemming from invidious upward social comparisons. Additionally, people who are lower in social status may appraise an event differently compared to those who occupy more prestigious positions, i.e. they are more likely to perceive something happening to them as unfair or as the result of an injustice [28–30]. In turn, frustration may give rise to aggression, expressed in the form of bullying behavior towards those who are lower down on the hierarchy [31]. Subjective social status (SSS), defined as “the individual’s perception of his/her own position in the social hierarchy” [32], has been found to predict health status independently of objective indicators of socioeconomic position such as educational attainment and income [33]. The extent to which SSS predicts health net of objective socioeconomic indicators is hypothesized to capture the impact of psychosocial frustration associated with being lower in the social hierarchy. We therefore sought to test the relationship between SSS and workplace bullying, conditional on each worker’s objective socioeconomic status.

To the best of our knowledge, no previous study has attempted to systematically or simultaneously examine the foregoing hypotheses about the social determinants of workplace bullying. To know the risk groups of being bullied would contribute to the prevention of workplace bullying. The aim of the present study was therefore to test whether: (1) characteristics related to workplace power distance (i.e., occupational status and employment type), (2) organizational characteristics related to safety climate (proxied by company/establishment size), and (3) SSS as an indicator of perceived social inequality or disadvantage, were related to the witnessing or personal experience of workplace bullying. We analyzed data from a nationally representative survey of the working population in Japan [34].

## Methods

### Participants

A questionnaire survey was administered to a nationally representative community-based sample of 5,000 residents in Japan aged 20–60 years from November 2010 to February 2011. A two-step random sampling procedure was adopted. First, all forty seven prefectures in Japan were grouped into 11 strata. The municipalities within each stratum were further grouped into 100 survey sites according to their population size (e.g., city with a population of more than 200,000, or city with a population of less than 200,000). A total of 5,000 individuals were randomly selected from the official residential registry at each survey site, and an invitation letter, questionnaire, and return envelope were mailed to each individual in November 2010. Those individuals who agreed to complete the survey did so anonymously. A total of 2,384 agreed to participate and completed the questionnaire (response rate, 47.7%). After excluding 751 respondents who were not active in the labor force at the time of the survey and 87 respondents who had missing responses on gender, age, education, household income, SSS, occupation, employment contract, company size, establishment size, or industry, the data from 1,546 respondents (809 men and 737 women) were analyzed.

### Ethics Statement

The Ethical Committee of the Graduate School of Medicine/Faculty of Medicine, the University of Tokyo reviewed and approved aims, and procedures of this study before conducting the survey (No. 2953).

### Measures

#### Workplace bullying

Workplace bullying was assessed by self-report, and we inquired about both the personal experience of bullying victimization as well as witnessing it in others [2,35]. Respondents were asked whether they personally experienced bullying during the past 30 days, using a single-item “Have you been bullied in your workplace?” The respondents who chose “yes” were defined as “victims”. In the survey, we did not present a definition of bullying to respondents due to limitations of space. In addition to this question, respondents were also asked whether they had witnessed bullying in their workplace during the past 30 days.

#### Social class indicators

Occupational status of respondents was grouped into the following five categories: managers (e.g., vice-president, division manager, or section chief, etc.), non-manual (e.g., engineer, technicians, teachers, physician, nurses, clerks, accountants, data-entry operators, salesclerks, merchandise selling profession, real-estate salespersons, etc.), service (e.g., service workers, hairdressers, waiters/waitresses, home helpers), manual (e.g., drivers, transporters, telephone operators, tool makers, assembly-line operators, carpenters, construction assistants, etc.), and others.

Employment type was grouped into the five categories: permanent, temporary employees, contract employees, part-time workers, and owner/executive officer/others.

The industrial group classification was based on the Japan Standard Industry Classification (JSIC) but the number of participants within each classification was too small so that primary, secondary, and tertiary sector categories were used for analysis purpose [36]. Company size and establishment size was categorized based on the Industrial Safety and Health Law in Japan.

Education was measured using a single-item question; “What level of educational attainment have you completed?” The respondents were asked to choose the following options: primary/junior high school, high school, vocational school, junior college, university, and graduate school. In our analysis, the levels of education were combined into four categories: junior high school graduate, high school graduate, vocational school/college graduate, and university graduate or higher.

Annual household income over the preceding year was also measured using a single-item question; “What was the sum of earnings for your whole household over the past one year?” The respondents were asked to choose one of the following options: less than one million yen (US$11,000, if $1 = 110 yen), 1.00–2.49 million yen (US$11,000–27,390), 2.50–4.99 million yen (US$27,390–54,890), 5.00–7.49 million yen (US$54,890–82,390), 7.50–9.99 million yen (US$82,390–109,890), over 10.0 million yen (US$110,000), or unknown. Since the number of respondents in the “less than one million yen” stratum category was small (n = 18), “less than one million yen” and “1.00–2.49 million yen” categories were combined into “less than 2.5 million yen” category for analysis purpose. The average household income in Japan was 5,345,000 yen (US$58,795) and the median was 4,270,000 yen (US$46,970) in 2009 [37] so that respondents who reported less than 2.5 million yen (US$27,500) income represent a level of income that is below approximately half of the median household income (the conventional definition of poverty in the OECD).

SSS was measured using a single-item question developed by Sakurai et al. [38]. The respondents were asked, “If Japanese society was divided into 10 social strata, which stratum do you suppose your household would belong to?” Although the SSS scale used in this study was not the one that is used most widely for research on SSS, i.e., the MacArther Scale of Subjective Social Status in a ladder format with 10 steps [33], the scale used in this study avoided the use of terms such as “class,” “income,” or “education” when asking respondents to rate their SSS. Since the number of respondents in the extreme categories was small, the respondents who chose the lowest (n = 36) and the second lowest (n = 89) strata were combined into the “lower” category, while the third (n = 212) and the fourth (n = 249) were combined into “lower middle”, the fifth (n = 420) and the sixth (n = 313) were combined into “middle”, and the seventh (n = 176), eighth (n = 40), ninth (n = 8), and top strata (n = 3) were combined into “upper/upper middle”.

### Statistical analysis

We used multiple logistic regression analysis to examine the relationship between each SES indicator and workplace bullying. Odds ratios (ORs) and 95% Confidence Intervals (CIs) were calculated adjusting for demographic variables (gender and age) (Model 1), work-related characteristics (occupational status, employment type, industrial grouping, company size, and establishment size) and non-work related SES (education, household income) (Model 2), and SSS (Model 3). The 2-tailed *p* value for statistical significance to see the differences among each social indicator was set at 0.05. All analyses were conducted using SPSS 21.0 for Windows.

## Results

### Participant characteristics

Approximately half of the respondents were non-manual workers, and 20% and 10% of respondents were manual and service workers, respectively. Over 60% of respondents were permanent workers, while 20% were part-time workers. About 30% of respondents worked at companies with fewer than 50 employees, while another 30% worked for in the civil service. Seventy percent of respondents worked in the tertiary sector, while less than one percent was engaged in the primary sector. Although the limited information on demographic characteristics of general working population in Japan, ratios of gender, employment contract, company size, and industrial groups in our data were are broadly comparable to general working population reported on Annual Report on the Labor Force Survey [39].

Three out of ten respondents were university graduates or higher, while a further 30% were vocational school/junior college graduates, and the rest were high school graduates (Table 1). Among 10% of respondents annual household income was more than 9.99 million yen (US$109,890), while 8% of respondents reported incomes lower than 2.5 million yen (US$27,500). About half of the respondents placed themselves in the middle stratum of SSS, and one third in the lower middle.

**Table 1:** Characteristics of respondents (*N* = 1,546).

	n	%		n	%
Gender			Company size		
Male	809	52.3	<50	419	27.1
Female	737	47.7	50–299	367	23.8
Age group			300–999	225	14.6
>50	425	27.5	>999	427	27.6
40–49	441	28.5	Civil service	108	7.0
30–39	435	28.1	Establishment size		
<30	245	15.8	<50	840	54.3
Education			50–299	457	29.6
University/graduate school graduate	431	27.9	300–999	139	9.0
Vocational school/college graduate	424	27.4	>999	110	7.1
High school graduate	611	39.5	Industrial groups		
Junior high school graduate	80	5.2	Tertiary sector †		
Household income (yen per year)			Telecommunication	55	3.6
>9.99 million	157	10.2	Transport	76	8.5
7.5–9.99 million	250	16.2	Wholesale and retail trade	164	10.6
5.0–7.49 million	416	26.9	Finance and insurance	74	4.8
2.5–4.99 million	469	30.3	Letting and sale of real estate	10	0.6
<2.5 million	126	8.2	Research study and consulting business	18	1.2
Unknown	128	8.3	Hotels, restaurants and entertainment	64	4.1
Subjective socioeconomic status (SSS)			Education and learning assistance	66	4.3
Upper/upper middle (7–10)	227	14.7	Healthcare and welfare	212	13.7
Middle (5–6)	733	47.4	Other service industries	151	9.6
Lower middle (3–4)	461	29.8	Public administration	100	6.5
Lower (1–2)	125	8.1	Others	102	6.6
Occupations			Secondary sector		
Managers	151	9.8	Construction	99	6.4
Non-manual workers	803	51.9	Manufacturing	313	20.2
Service workers	155	10.0	Electricity, gas and water supply	32	2.1
Manual workers	302	19.5	Primary sector		
Others	135	8.7	Agriculture, forestry and fishing	8	0.5
Employment contract			Mining and quarrying	2	0.1
Permanent	1,014	65.6			
Temporary employees	36	2.3	Workplace bullying		
Contract employees	94	6.1	Victims	94	6.1
Part-time workers	350	22.6	Bystanders	229	14.8
Owner/executive officer/others	52	3.4			

† Primary, secondary and tertiary sector were categorized according to Clark, 1940.

A total of 94 (6.1%) respondents reported personally experiencing workplace bullying and 229 (14.8%) respondents witnessed workplace bullying.

### Association of social class indicators with experiencing workplace bullying

There was no significant association between gender and experiencing bullying, while the prevalence among those who were younger than thirty years old was higher than among older workers (*p* = 0.021, see Table 2). After adjusting for gender and age, temporary employees were more likely to report workplace bullying compared to permanent employees (OR = 2.62, 95%CI = 1.01–6.79, see Table 3). The odds ratio of experiencing bullying was also significantly elevated for junior high school graduates compared to university/graduate school graduates (OR = 2.45, 95%CI = 1.03–5.85). The prevalence of workplace bullying was significantly elevated in the lower household income groups and in the lower SSS stratum. After additionally adjusting for work-related characteristics (occupational status, employment type, industrial grouping, company size, and establishment size) and non-work related SES (education, household income), we found higher odds of experiencing bullying among respondents with less than 2.5 million yen (US$27,500) annual household income (OR = 4.24, 95%CI = 1.48–12.1, Model 2). After all variables were simultaneously entered in the model, household income and SSS remained significantly associated with workplace bullying (*p* = 0.017 and 0.002, respectively, Model 3). The associations of occupational status, company size, establishment size, and industry were not significant in any model (*p* > 0.05).

**Table 2:** The prevalence and odds ratio for experience of workplace bulling by social class indicators among representative samples of Japanese workers (N = 1,546).

	n (Victims)	n (All)	Prevalence (%)	Model 1 (95%Cl)	Model 2 (95%Cl)	Model 3 (95%Cl)
Gender						
Male	47	809	5.8	―	1.00	1.00
Female	47	737	6.4	―	1.02 (0.59 to 1.76)	1.13 (0.65 to 1.95)
			p = 0.641	―	p = 0.947	p = 0.669
Age group						
>50	19	425	4.5	―	1.00	1.00
40–49	28	441	6.3	―	1.49 (0.79 to 2.81)	1.44 (0.76 to 2.72)
30–39	22	435	5.1	―	1.17 (0.58 to 2.35)	1.09 (0.54 to 2.19)
<30	25	245	10.2	―	2.12 (1.03 to 4.34)*	1.83 (0.89 to 3.75)
			p = 0.021	―	p = 0.144	p = 0.265
Education						
University/graduate school graduate	23	431	5.3	1.00	1.00	1.00
Vocational school/college graduate	33	424	7.8	1.55 (0.87 to 2.76)	1.45 (0.80 to 2.65)	1.40 (0.77 to 2.56)
High school graduate	30	611	4.9	1.02 (0.58 to 1.81)	0.98 (0.52 to 1.84)	0.90 (0.48 to 1.69)
Junior high school graduate	8	80	10.0	2.45 (1.03 to 5.85) *	2.13 (0.79 to 5.76)	1.88 (0.70 to 5.08)
			p = 0.109	p = 0.081	p = 0.191	p = 0.208
Household income (yen per year)						
>9.99 million	6	157	3.8	1.00	1.00	1.00
7.5–9.99 million	18	250	7.2	1.91 (0.74 to 4.95)	1.90 (0.71 to 5.03)	1.74 (0.64 to 4.73)
5.0–7.49 million	13	416	3.1	0.84 (0.31 to 2.27)	0.91 (0.33 to 2.52)	0.70 (0.24 to 2.02)
2.5–4.99 million	27	469	5.8	1.49 (0.60 to 3.70)	1.52 (0.57 to 4.04)	0.91 (0.33 to 2.54)
<2.5 million	18	126	14.3	4.13 (1.58 to 10.8) **	4.24 (1.48 to 12.1) **	2.34 (0.78 to 7.01)
Unknown	12	128	9.4	2.22 (0.79 to 6.24)	2.02 (0.68 to 5.96)	1.44 (0.48 to 4.37)
			p < 0.001	p = 0.001	p = 0.004	p = 0.017
Subjective socioeconomic status (SSS)						
Upper/upper middle (7–10)	7	227	3.1	1.00	1.00	1.00
Middle (5–6)	30	733	4.1	1.30 (0.56 to 3.02)	―	1.49 (0.61 to 3.64)
Lower middle (3–4)	41	461	8.9	2.92 (1.28 to 6.67) *	―	3.43 (1.34 to 8.75) *
Lower (1–2)	16	125	12.8	4.21 (1.66 to 10.7)**	―	4.57 (1.59 to 13.1) **
			p < 0.001	p < 0.001	―	p = 0.002

* p<0.05

** p<0.01

Model 1: gender and age adjusted.

Model 2: all variables except SSS were simultaneously entered in the model.

Model 3: all variables were simultaneously entered in the model.

**Table 3:** The prevalence and odds ratio for experience of workplace bulling by social class indicators among representative samples of Japanese workers (cont.) (N = 1,546).

	n (victims)	n (all)	Prevalence (%)	Model 1 (95%Cl)	Model 2 (95%Cl)	Model 3 (95%Cl)
Occupational status						
Non-manual workers	47	803	5.9	1.00	1.00	1.00
Service workers	8	155	5.2	0.88 (0.40 to 1.92)	0.86 (0.38 to 1.95)	0.82 (0.36 to 1.87)
Manual workers	20	302	6.6	1.32 (0.77 to 2.35)	1.40 (0.69 to 2.85)	1.32 (0.65 to 2.69)
Managers	8	151	5.3	1.22 (0.52 to 2.84)	1.57 (0.63 to 3.91)	1.88 (0.75 to 4.41)
Others	11	135	8.1	1.58 (0.79 to 3.16)	1.57 (0.75 to 3.30)	1.54 (0.73 to 3.25)
			p = 0.803	p = 0.642	p = 0.583	p = 0.501
Employment status						
Permanent	61	1,014	6.0	1.00	1.00	1.00
Temporary employees	6	36	16.7	2.62 (1.01 to 6.79)*	1.84 (0.68 to 5.00)	1.67 (0.61 to 4.56)
Contract employees	8	94	8.5	1.54 (0.70 to 3.39)	1.24 (0.54 to 2.83)	1.13 (0.50 to 2.59)
Part-time workers	17	350	4.9	0.82 (0.44 to 1.53)	0.61 (0.31 to 1.22)	0.57 (0.28 to 1.12)
Owner/executive officer/others	2	52	3.8	0.69 (0.16 to 2.91)	0.51 (0.11 to 2.49)	0.55 (0.11 to 2.67)
			p = 0.072	p = 0.165	p = 0.253	p = 0.268
Company size						
<50	26	419	6.2	1.00	1.00	1.00
50–299	20	367	5.4	0.86 (0.47 to 1.57)	0.90 (0.46 to 1.77)	0.85 (0.43 to 1.69)
300–999	13	225	5.8	0.87 (0.43 to 1.73)	0.96 (0.45 to 2.04)	0.91 (0.42 to 1.96)
>999	31	427	7.3	1.12 (0.65 to 1.94)	1.41 (0.71 to 2.80)	1.36 (0.68 to 2.72)
Civil service	4	108	3.7	0.61 (0.21 to 1.78)	0.72 (0.22 to 2.36)	0.80 (0.24 to 2.65)
			p = 0.668	p = 0.750	p = 0.561	p = 0.618
Establishment size						
<50	54	840	6.4	1.00	1.00	1.00
50–299	24	457	5.3	0.83 (0.51 to 1.37)	0.86 (0.48 to 1.53)	0.90 (0.51 to 1.62)
300–999	10	139	7.2	1.08 (0.53 to 2.19)	1.54 (0.47 to 2.35)	1.13 (0.50 to 2.54)
>999	6	110	5.5	0.80 (0.33 to 1.91)	0.73 (0.28 to 1.94)	0.80 (0.30 to 2.14)
			p = 0.777	p = 0.838	p = 0.879	p = 0.911
Industrial groups						
Tertiary sector	71	1,092	6.5	1.00	1.00	1.00
Secondary sector	22	444	5.0	0.79 (0.47 to 1.32)	0.67 (0.36 to 1.24)	0.66 (0.35 to 1.22)
Primary sector	1	10	10.0	1.68 (0.21 to 13.6)	1.24 (0.13 to 11.9)	1.57 (0.17 to 14.9)
			p = 0.454	p = 0.574	p = 0.425	p = 0.360

* p<0.05 ** p<0.01

Model 1: gender and age adjusted.

Model 2: all variables except SSS were simultaneously entered in the model.

Model 3: all variables were simultaneously entered in the model.

### Association of social class indicators with witnessing workplace bullying

We found no significant association between gender, age, and witnessing bullying (Table 4). After adjusting for gender and age, employment contract type was significantly associated with witnessing bullying at work, with temporary employees having a high odds ratio (OR = 2.70, 95%CI = 1.27–5.73, see Table 5). For occupational status, managers reported the lowest odds of witnessing bullying (OR = 0.52, 95%CI = 0.28–0.99) compared to non-manual workers. In terms of non-work related SES, the prevalence of witnessing workplace bullying varied significantly among groups classified according to education, household income, and SSS (*p* = 0.017, 0.030, and 0.000, respectively, Model 1 in Table 4). Junior high school graduates had the highest odds (OR = 2.26, 95%CI = 1.26–4.03) compared to university/graduate school graduates. The lower household income groups (OR = 3.25, 95%CI = 1.60–6.62) and lower SSS groups (OR = 3.67, 95%CI = 1.91–7.07) also had higher odds ratios of witnessing bullying at work. After additionally adjusting for work-related characteristics (occupational status, employment type, industrial grouping, company size, and establishment size) and non-work related SES (education, household income) (Model 2), ORs were significantly elevated for witnessing bullying among temporary employees (OR = 2.32, 95% CI = 1.08–5.03) and those reporting less than 2.5 million yen (US$27,500) annual household income (OR = 2.62, 95%CI = 1.22–5.63). After all variables were simultaneously entered in the model (Model 3), only SSS remained significantly associated with witnessing workplace bullying (*p* = 0.017). Education and industry were marginally significantly associated with witnessing bullying in a nonlinear manner, with the vocational school or college graduates and primary sector group at a higher risk (*p* = 0.051 and *p* = 0.052, respectively). Company size and establishment size were not associated with witnessing bullying in any model (*p* > 0.05). When we repeated the same analyses restricted only to workers who did not personally experience workplace bullying (n = 1,452), the results obtained were essentially unchanged.

**Table 4:** The prevalence and odds ratio for witnessing workplace bullying by social class indicators among representative samples of Japanese workers (N = 1,546).

	n (victims)	n (all)	Prevalence (%)	Model 1 (95%Cl)	Model 2 (95%Cl)	Model 3 (95%Cl)
Gender						
Male	125	809	15.5	―	1.00	1.00
Female	104	737	14.1	―	0.90 (0.61 to 1.31)	0.94 (0.64 to 1.37)
			p = 0.899	―	p = 0.572	p = 0.732
Age group						
>50	57	425	13.4	―	1.00	1.00
40–49	69	441	15.6	―	1.15 (0.77 to 1.71)	1.13 (0.76 to 1.69)
30–39	65	435	14.9	―	1.00 (0.66 to 1.52)	0.97 (0.64 to 1.47)
<30	38	245	15.5	―	1.01 (0.62 to 1.67)	0.93 (0.56 to 1.53)
			p = 0.801	―	p = 0.872	p = 0.810
Education						
University/graduate school graduate	63	431	14.6	1.00	1.00	1.00
Vocational school/college graduate	65	424	15.3	1.04 (0.74 to 1.64)	0.97 (0.64 to 1.46)	1.50 (0.78 to 2.88)
High school graduate	80	611	13.1	0.93 (0.64 to 4.03)	0.75 (0.50 to 1.12)	0.72 (0.48 to 1.07)
Junior high school graduate	21	80	26.3	2.26 (1.26 to 4.03) **	1.62 (0.84 to 3.11)	0.94 (0.62 to 1.42)
			p = 0.024	p = 0.017	p = 0.050	p = 0.051
Household income (yen per year)						
>9.99 million	13	157	8.3	1.00	1.00	1.00
7.5–9.99 million	33	250	13.2	1.65 (0.84 to 3.24)	1.48 (0.74 to 2.97)	1.32 (0.65 to 2.66)
5.0–7.49 million	59	416	14.2	1.80 (0.96 to 3.40)	1.63 (0.84 to 3.15)	1.31 (0.66 to 2.58)
2.5–4.99 million	76	469	16.2	2.13 (1.14 to 3.97) *	1.72 (0.89 to 3.35)	1.22 (0.61 to 2.45)
<2.5 million	28	126	22.2	3.25 (1.60 to 6.62) **	2.62 (1.22 to 5.63) *	1.74 (0.78 to 3.88)
Unknown	20	128	15.6	2.12 (0.66 to 4.53)	1.66 (0.76 to 3.66)	1.30 (0.58 to 2.90)
			p = 0.037	p = 0.030	p = 0.249	p = 0.772
Subjective socioeconomic status (SSS)						
Upper/upper middle (7–10)	17	227	7.5	1.00	1.00	1.00
Middle (5–6)	99	733	13.5	1.96 (1.14 to 3.36)*	―	1.72 (0.97 to 3.06)
Lower middle (3–4)	85	461	18.4	2.82 (1.62 to 4.90) **	―	2.30 (1.24 to 4.23) **
Lower (1–2)	28	125	22.4	3.67 (1.91 to 7.07)**	―	2.98 (1.44 to 6.17) **
			p < 0.001	p < 0.001	―	p = 0.017

* p<0.05

** p<0.01

Model 1: gender and age adjusted.

Model 2: all variables except SSS were simultaneously entered in the model.

Model 3: all variables were simultaneously entered in the model.

**Table 5:** The prevalence and odds ratio for witnessing workplace bullying by social class indicators among representative samples of Japanese workers (cont.) (N = 1,546).

	n (victims)	n (all)	Prevalence (%)	Model 1 (95%Cl)	Model 2 (95%Cl)	Model 3 (95%Cl)
Occupational status						
Non-manual workers	119	803	14.8	1.00	1.00	1.00
Service workers	22	155	14.2	0.98 (0.60 to 1.62)	1.00 (0.59 to 1.69)	0.97 (0.57 to 1.64)
Manual workers	58	302	19.2	1.33 (0.92 to 1.92)	1.37 (0.88 to 2.14)	0.33 (0.85 to 2.09)
Managers	13	151	8.6	0.52 (0.28 to 0.99) *	0.66 (0.34 to 1.27)	0.70 (0.36 to 1.37)
Others	17	135	12.6	0.84 (0.48 to 1.44)	0.82 (0.46 to 1.46)	0.79 (0.44 to 1.40)
			p = 0.044	p = 0.058	p = 0.265	p = 0.351
Employment status						
Permanent	151	1,014	14.9	1.00	1.00	1.00
Temporary employees	11	36	30.6	2.70 (1.27 to 5.73) *	2.32 (1.08 to 5.03) *	2.20 (1.02 to 4.79) *
Contract employees	16	94	17.0	1.24 (0.70 to 2.21)	1.09 (0.60 to 1.98)	1.06 (0.58 to 1.92)
Part-time workers	48	350	13.7	1.03 (0.68 to 1.55)	0.90 (0.57 to 1.41)	0.87 (0.56 to 1.37)
Owner/executive officer/others	3	52	5.8	0.36 (0.11 to 1.17)	0.41 (0.12 to 1.45)	0.46 (0.13 to 1.62)
			p = 0.036	p = 0.035	p = 0.119	p = 0.172
Company size						
<50	57	419	13.6	1.00	1.00	1.00
50–299	60	367	16.3	1.23 (0.83 to 1.83)	1.28 (0.82 to 2.01)	1.27 (0.81 to 2.00)
300–999	33	225	14.7	1.07 (0.67 to 1.83)	1.28 (0.76 to 2.14)	1.27 (0.76 to 2.13)
>999	65	427	15.2	1.13 (0.78 to 1.66)	1.39 (0.85 to 2.25)	1.36 (0.84 to 2.21)
Civil service	14	108	13.0	0.94 (0.50 to 1.77)	1.21 (0.59 to 2.50)	1.30 (0.63 to 2.68)
			p = 0.823	p = 0.837	p = 0.745	p = 0.787
Establishment size						
<50	124	840	14.8	1.00	1.00	1.00
50–299	72	457	15.8	1.07 (0.78 to 1.47)	0.99 (0.68 to 1.44)	0.99 (0.68 to 1.45)
300–999	15	139	10.8	0.68 (0.38 to 1.20)	0.67 (0.36 to 1.26)	0.67 (0.35 to 1.25)
>999	18	110	16.4	1.09 (0.64 to 1.88)	1.05 (0.56 to 1.98)	1.08 (0.58 to 2.04)
			p = 0.514	p = 0.500	p = 0.596	p = 0.572
Industrial groups						
Tertiary sector	71	1,092	6.5	1.00	1.00	1.00
Secondary sector	22	444	5.0	1.03 (0.74 to 1.42)	0.90 (0.61 to 1.33)	0.90 (0.61 to 1.33)
Primary sector	1	10	10.0	3.90 (1.09 to 14.0)*	4.48 (1.16 to 17.3) *	5.10 (1.29 to 20.1) *
			p = 0.454	p = 0.114	p = 0.074	p = 0.052

* p<0.05

**p<0.01

Model 1: gender and age adjusted.

Model 2: all variables except SSS were simultaneously entered in the model.

Model 3: all variables were simultaneously entered in the model.

## Discussion

The present study found that low SSS was significantly and positively associated with experiencing and witnessing workplace bullying, independently of other work characteristics and non-work related SES indicators, supporting a frustration hypothesis. Temporary employees reported significantly higher prevalence of witnessing workplace bullying compared to permanent employees, while other non-regular employees did not report an increase. This finding partially supports the power-distance hypothesis. While educational status and household income was significantly and inversely associated with workplace bullying prior to adjusting for SSS, they became non-significant after adjusting for SSS. Our results do not support the organizational safety culture hypothesis, although we hasten to add that we did not directly assess safety climate but instead used a proxy indicator (company size). Taken together, SSS showed the strongest association with both the personal experience of and witnessing bullying.

One possible explanation for this finding is that SSS is better at capturing—over and above education, occupational status and income—the psychological sense of insecurity or shame associated with being lower in the societal hierarchy. SSS is inherently relational, i.e. it describes an individual’s position as being higher to lower *relative* to other people around them. Hence, even in a relatively homogeneous context—for example, in a workplace where everyone has a similar degree of educational attainment or earns a similar income—there can develop a social hierarchy based upon perceived status. Lower status in this sense seems to be a stronger predictor of bullying victimization compared to traditional (and objective) indicators of socioeconomic status.

An alternative explanation for our finding is that individuals who perceived themselves to be lower in the social hierarchy are more likely to perceive an event occurring to them as stemming from injustice or unfair treatment [28–30]. In the present study, we did not attempt to provide a definition of bullying in the questionnaire, and thus all reports of experiencing or witnessing bullying are via self-report. It is possible that individuals with low SSS are more likely to experience or witness workplace bullying. Because SSS is also self-perceived, this may have contributed to common source bias.

Temporary employees ("haken shain") were more likely to experience and observe workplace bullying compared to permanent workers in this study, whereas occupational status was not associated with workplace bullying. One of the possible explanations is that temporary employment is the strongest predictor of being in a lower position in an organization because they lack decision-making authority [16,17]. This is compatible with studies that reported high prevalence among workers occupying the lowest position in the organization such as unskilled workers [12,13]. Another possible explanation is job insecurity. Temporary employment is characterized by lack of protection from labor laws (such as anti-discrimination legislation) and hence temporary employees may be especially vulnerable to social exclusion in the workplace [16,17,40]. Especially in a group-oriented culture such as Japanese society, not being received into the group may make people feel insecure [18]. A typical illustration of this social exclusion is the practice (by permanent workers) of calling a temporary employee “haken-san” (“haken” means a temporary employee and “san” means Mr. or Ms. in Japanese), not using his/her name. We also found that temporary employees were significantly more likely to witness bullying even when we restricted and re-ran the analyses only among workers who did not experience workplace bullying. This indicates temporary employees work in poor working environments that create and sustain conditions that are conducive to bullying [24]. The present findings partly support our power distance hypothesis and expanded this evidence to the working population in Japan.

The present study found that employees who received no high school education and had the lowest annual household incomes (< 2.5 million yen or US$27,500) were at highest risk not only for experiencing but also witnessing bullying. Although few reports are available on the relation between non-work related SES indicators and workplace bullying, the results in this study are in line with previous research suggesting an association between socioeconomic disadvantage and bullying in childhood [41], and among school-aged adolescents [42] as well as lifetime bullying behavior toward others [43]. As SES is reported as an indicator of psychosocial work environment [44], the findings in this study also suggests that non-work SES could be an indicator of working at poor working environments which leads to bullying.

In the present study, company and establishment size were not associated with experiencing and witnessing workplace bullying. Although this result tends not to support our hypothesis about safety climate, we hasten to add that company size is an imperfect proxy of organizational culture, i.e. not every large company makes the same degree of investment or commitment towards maintaining a safe workplace climate [23].

The overall reported prevalence of workplace bullying in the current study was six percent. Fifteen percent had witnessed colleagues being bullied. These figures are lower compared to other studies in Japan (9.0% to 15.4%) [3–5] as well as the average prevalence rate reported in a meta-analysis [2]. It is well known that prevalence of bullying is highly dependent on the measurement method. For example, in the previous study when using a self-labeling method with a definition of bullying the prevalence was lower (5.9%) than when using a behavioral experience method, which asked respondents whether they had experienced various negative acts during the past six months (9.0%) using the same dataset [5]. The prevalence rates mentioned above [2–7] were assessed by a behavioral experience method, while the measurement used in this study was a self-labeling method without a definition of bullying and asked about current experience at the time of the survey, which may have contributed to the low prevalence found.

### Limitations

Several limitations of the present study should be noted. First, the study is cross-sectional, so we cannot draw causal influences regarding whether social class indicators leads to experiencing workplace bullying, or experiencing bullying determine his/her social class. However, in this study bullying was also correlated with other objective indicators of socioeconomic position, such as temporary work status, educational attainment, and household income, which are unlikely to have been affected by reverse causation. Thus, we assume that SES/SSS is an indicator of bullying but longitudinal studies are needed to clarify this causality. Secondly, we used self-report measures and there may have been issues with self-reporting bias, especially in terms of experiencing workplace bullying. Although we used two methods to measure bullying in this study to capture a more accurate picture of workplace bullying, there is a possibility that individuals with different SSS may have different definition and understanding of bullying. In addition, measuring the thirty-day prevalence of workplace bullying may contribute to underestimation. We also did not inquire about perpetration of bullying so that we could not identify whether bullying occurred among workers from similar SES backgrounds, or whether it was directed by higher SES workers toward lower SES workers. Lastly, some psychological states such as negative affectivity may have affected both perceptions of being bullied and self-perception of social status [38,45].

## Conclusions

The prevalence of workplace bullying varies by not only employment type but also by non-work related socioeconomic indicators such as education, household income, and SSS. Specifically, the current investigation found that younger employees who received no high school education, worked as a temporary employee, had low annual household incomes, and lower subjective social status were at highest risk for experiencing workplace bullying. Future prevention of workplace bullying should focus on workers with low SES or SSS.
